# Using improved climate forecasting in cash crop planning

**DOI:** 10.1186/2193-1801-3-422

**Published:** 2014-08-10

**Authors:** Ramya Rachmawati, Melih Ozlen, John W Hearne, Yuriy Kuleshov

**Affiliations:** School of Mathematical and Geospatial Sciences RMIT University, Melbourne, Australia; Mathematics Department Faculty of Mathematics and Natural Sciences, University of Bengkulu, Bengkulu, Indonesia; Bureau of Meteorology, GPO Box 1289, Melbourne, Vic 3001 Australia

**Keywords:** Climate forecast, Agriculture, Mathematical programming, Crop planning

## Abstract

**Abstract:**

Developments in meteorology over the last couple of decades have enabled significant improvements to be made in the accuracy of seasonal forecasts. This paper focuses on developing a model for cash crop planning that utilises these forecasts. It does this by determining the rate of growth of each crop as a function of heat units accumulated. This enables time to maturity to be determined and used in planning, particularly for planting new crops, removing unprofitable immature crops, and harvesting mature crops for profits. The proposed model is solved on a rolling horizon basis. To illustrate the advantage to be gained from improved seasonal forecasts the model is first applied to a problem using long-term temperature averages (climatology). Solutions to the same problem utilising improved seasonal forecasts for temperature are then obtained. This forecast proves to be a valuable input to the model and makes the second approach outperform the first consistently in our simulations.

## 1 Introduction

Since the early work of (Phillips 1956), models to forecast monthly and seasonal patterns of weather continue to improve, (Lynch 2006). Thus the observation by Chen and McCarl (2000) is pertinent “.. researchers are involved in an effort to determine whether systematic disturbances in climate can be detected and exploited in terms of improved decision making which is conditional on climate information”. In this paper we consider the crop planning problem and formulate the model that enables the advantages to be gained from more accurate improved weather forecasts that are available for longer periods of time compared to decades ago.

In the crop planning problem, farmers have to decide on what and when to plant to maximise their profits. They often rely on planting calendars to decide the best time for planting certain crops (FAO 2014). However, these calendars assume static growth rates based only on long-term average weather data. They do not take into account the latest monthly and seasonal forecasts of temperature and rainfall. In practice, however, the crops’ time to maturity may change because of the weather conditions, as reported by Schlenker and Roberts (2009). In fact this effect was first observed several decades ago by Boswell (1929) in a study of peas. Thus to fully exploit improved seasonal weather forecasts we adopt the concept of 'heat’ or 'thermal’ units often expressed in degree-days (DD) used in most modern crop simulation models. The rate at which many biological processes occur increases linearly with temperature above a certain threshold. Below this threshold temperature no development or maturation takes place. Thus the time taken for a crop to be ready for harvesting from the time it was sown depends on the accumulated heat units. This is the integral over that period of a linear function of temperature (Snyder 1985). Of course this assumes that the temperature is always below some upper threshold of tolerance (eg. fire will destroy a plant!).

In this work we consider a case where water requirements are met by a good irrigation system and we assume that solar exposure of each crop does not deviate significantly from that already considered in the planting calendar. However, rainfall and solar exposure could be incorporated into our model with similar formulations to those used for temperature.

Linear programming has been successfully applied to a wide range of problems in the agricultural sector (Weintraub and Romero 2006). Clarke (1989) uses this approach to determine strategies to maximise farmers’ profits. Clarke’s model, which considers diversification and crop rotation, was applied to a crop selection problem in Bangladesh. The model uses constant growth rates based on long-term averaged climatic data. In contrast, we propose a linear programming model for crop planning with growth rates dependent on accumulated heat units. The accumulated heat units are based on temperature forecasts. Improved seasonal weather forecasts and hence more accurate time to maturity estimates, enables improved planning, particularly relating to the planting of new crops, removing unprofitable crops, and harvesting mature crops for profits. Similarly seasonal rainfall forecasts could be used but in this work we only consider irrigated cash crops. The model is capable of handling dynamic price for cash crops.

Stochastic programming and the rolling horizon approach are commonly used for making decisions under conditions of uncertainty. Darby-Dowman et al. (2000) propose two-stage stochastic programming with recourse to determine an optimal horticulture planting plan in Burkina Faso. However, two-stage stochastic programming is not considered in the model used in this paper, because there are no first stage decision variables that are binding in the longer term. Instead, the rolling horizon approach as discussed by Sethi and Sorger (1991) is used. By using this technique, decisions are regularly updated as more information becomes available. Thus at each time-step, an optimised schedule is generated about which new crops to plant, which mature crops to harvest, or which unprofitable immature crops to remove.

The mathematical model is presented in the next section. The model is then demonstrated on examples using a series of hypothetical data in Section 3. In Section 4, we conclude and discuss possible extensions of the model.

## 2 Model formulation

We consider a farm with a reliable and sufficient source of water that is suitable for growing several species of cash crops. At the beginning of each period after any mature crops are harvested, the decision of what crops to plant and whether to remove any unprofitable crops is made. This decision is based on the expected time to maturity of each crop and their price at that time. Calculation of the time from planting to harvesting is based on accumulated heat units which is determined from temperature forecasts as discussed in the previous section. In general each crop species will require different amounts of heat units before they are ready for harvest. Projected prices (and hence profits) are also needed to determine an optimal planting strategy. With the objective of maximising profit the following linear programming model is formulated.

Indices:

*i* = crop type, *i* = 1, 2, …, *I*

*t* = period, *t* = 0, 1, …

*h* = accumulated heat units (in degree-periods)

Parameters:

*H*_*i*_= heat units requirement for crop *i* to mature

*u*_*t*_= average accumulated heat units during period *t*

*s*_*i*,*h*,*t*_= profits per hectare of land from crop *i*, with accumulated heat units *h*, at time *t*

*A* = land availability (in hectares)

*P* = maximum allowable percentage of land a crop can occupy to restrict exposure to risk

Decision variables:

*x*_*i*,*h*,*t*_= area of land planted with crop *i*, with *h* units of heat, at time *t*

*R*_*i*,*h*,*t*_= harvested area of land of crop *i*, with *h* units of heat, at time *t*

profits:

max
1

subject to
2345

The objective function (1) maximises the total profits during a planning horizon. It is assumed that a crop has the full profits when it reaches its heat units requirement, which is *H*_*i*_. Some crops like carrots can be sold even when they are not fully mature while other crops are not marketable when removed before they fully mature.

Constraint (2) is the balance equation that keeps track of the heat units accumulated by each crop. This constraint gives us the option either to remove or to keep old crops. Constraint (3) ensures that crops will be harvested once they have accumulated sufficient heat units.

Constraint (4) states that for each period, the planting area should not exceed the available land.

Constraint (5) ensures that the area under crop *i* at time *t* does not exceed a prescribed percentage of the land available. For example, *P* = 0.25 implies that no single species of crop may occupy simultaneously more than 25% of the land. This implies that when the land is fully utilised there will be at least four different types of crops growing. By changing the right hand side value of this constraint, the model can also be used to create a risk-profit curve. The greater the value of *P*, the greater the profits are, and the higher the risk will be. By growing a variety of crops at any time, the risk against uncertainties may be reduced (dos Santos et al. 2011).

The model is solved at each time-step following the rolling horizon approach. The length of the planning horizon can be decided by the decision maker. Starting from fallow land, for example, at time = 0, information about heat unit requirements, crop prices, and expected temperature are used as inputs to solve the model. The output determines the strategy to maximise the total profits during the planning horizon. This optimal solution might suggest some immediate actions requiring certain crops to be planted at the beginning of time = 0. In some cases, no crops will be planted at that time. This is because the solution indicates that it will be more profitable to start planting some crops in later periods so that their expected harvest time will coincide with higher prices.

At time = 1, the model is re-solved. At this time, as input data the model uses not only expected future temperature and price information but also a realisation of the temperature during the previous period. When the previous period’s expected temperature and price information are different from the actual conditions, then the new optimal solution might require a crop to be removed prematurely unless, of course, there is sufficient fallow land still available. All crops are harvested and assumed sold when they have accumulated sufficient heat units.

## 3 An Australian case study

In this section, application of the model to an Australian case study is demonstrated. Temperature data were from the Australian Bureau of Meteorology National Temperature Outlook (BoM 2013) that issue frequent seasonal (three month) forecasts using the Predictive Ocean Atmosphere Model for Australia (Cottrill et al. 2013). Other data used in this example were obtained from AusVeg Domestic Industry Report (AusVeg 2013), and Edenseeds Planting Guide (Edenseeds 2010). The proposed model is used with two different data sets to enable comparison of the benefits to be gained by using the latest seasonal forecasts. In the first approach, the planning is made by using long-term average temperature and past temperature realisations, while in the second approach the planning utilises improved climate forecast information besides past temperature realisations.

Table 1 shows the earliest and latest time for planting the six candidate crops. The table also shows each crop’s heat units requirement. For example, from the table, we learn that potato needs 122 of heat units to reach maturity. Suppose a year is divided into 24-time periods. The earliest time for planting potato is in September (period 16), while the latest time for planting potato is in January (period 1).

**Figure 1:**
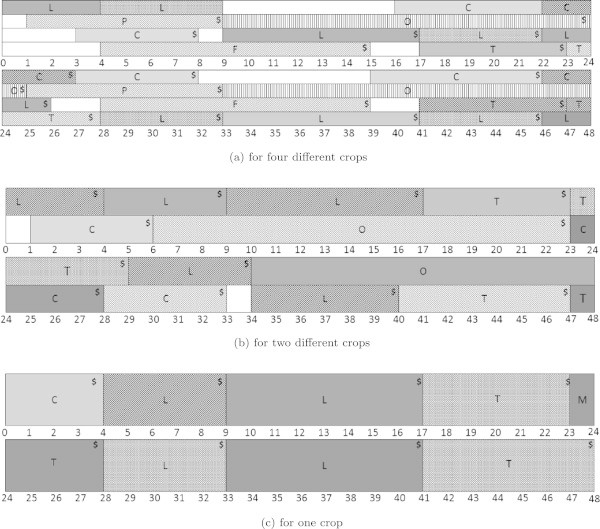
**Optimal solution.** P, T, O, C, L, F represent potatoes, tomatoes, onions, carrots, lettuces, and cauliflowers, respectively.

The model is solved using ILOG CPLEX 12.5.1 on a PC (Intel(R) Core(TM) i3 550 CPU 3.2 GHz processor and 4.0 GB RAM) with Python 2.7 programming language. Suppose four different crops may be planted at the same time. The solution gives us the order for planting crops to maximise profit in a long-term planning (normal) scenario, as can be seen in Figure 1a. We assume that planting and harvesting take place at the beginning of each period.

**Table 1:** **Planting calendar**

Name of crop	Earliest time	Latest time	Heat units requirement
	for planting	for planting	(degree-periods)
Potato	16	1	122
Tomato	16	23	90
Onion	2	17	210
Carrot	16	3	75
Lettuce	16	9	73
Cauliflower	18	7	130

The model is run 48 times, with the length of each planning horizon being two years. Starting from the fallow land at time =0, the immediate decisions are to plant lettuces. The crop then starts growing. When the realisation of temperature is the same as previously predicted, the lettuces will be harvested in the next 4 periods, when the heat units requirement is reached. However, the realisation may differ from the long-term conditions. In the next period, time = 1, we solve the model again by using the long-term average temperature and past temperature realisation.

Once a crop reaches its heat unit requirement, it is harvested and assumed sold. Figure 1b describes the optimal solution when we are allowed to plant two different crops at the same time, while Figure 1c represents the optimal solution when only one crop can grow at a time.

**Figure 2:**
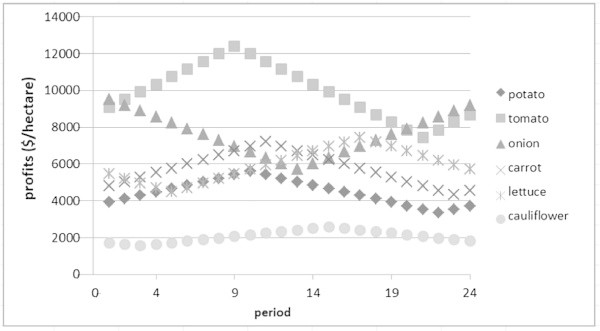
**Profits during a year.**

**Figure 3:**
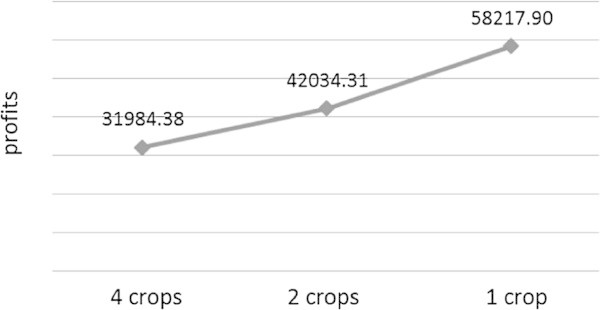
**The risk-profits curve for growing a number of different crops at any time.**

Profits may be affected by realisation of temperature and price. If the realisation of the temperature conditions and the price are exactly the same as displayed in Table 2 and Figure 2, then when we are required to grow four different crops at the same time, the profits of $31984.38 will be obtained for two years. For growing two different crops, the profits of $42034.31 will be obtained, while for growing one crop, the profits will be $58217.9 This results can be described in the risk-profits curve as can be seen in Figure 3. However, based on the realisation of temperature and price data, the planting time as well as the harvesting time may change, which in turn will affect the profits.

**Table 2:** **Temperature data used**

	Jan		Feb		Mar		Apr		May		Jun	
Period	0	1	2	3	4	5	6	7	8	9	10	11
Mean	19.8	19.8	19.8	18.9	18.1	16.5	14.9	13.4	11.9	10.8	9.8	9.4
	**Jul**		**Aug**		**Sep**		**Oct**		**Nov**		**Dec**	
Period	12	13	14	15	16	17	18	19	20	21	22	23
Mean	9.1	9.5	9.9	10.7	11.6	12.6	13.6	14.7	15.8	16.9	18.1	18.9

The comparison of the objective values resulting from both approaches is presented as follows. For the first approach, we update the solution once in 15 days using long-term (climatology) information, while for the second approach, the solution is always updated once in 15 days using new climate forecasting.

**Figure 4:**
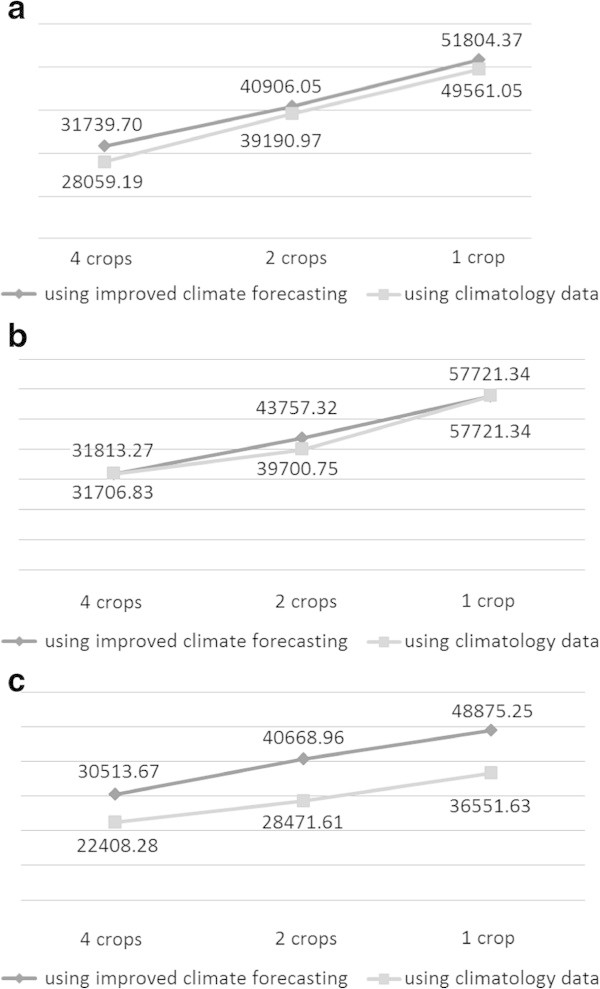
**Risk-profits curve.**

There are three scenarios of different temperature conditions. These are average temperature with higher variation, a warmer scenario and a cooler scenario. Table 3 represents the data of the realisation of temperature conditions for the high variation scenario. For the warmer and cooler scenarios, the temperature realisations are 5% higher and 5% lower than the average temperature represented in Table 2. We use data in Figure 2 for the three scenarios. The optimal solution of both approaches for the first, second, and third scenarios are summarised in Figure 4a, 4b and 4c.

**Table 3:** **Temperature realisation for the high variation scenario**

Period	0	1	2	3	4	5	6	7	8	9	10	11
Temperature	22.3	18.1	20.4	19.7	17.5	17.9	12.9	14.1	11.9	9.3	10.0	9.9
Period	12	13	14	15	16	17	18	19	20	21	22	23
Temperature	8.2	9.6	9.7	12.1	12.0	11.0	11.9	13.7	14.0	17.0	18.4	21.2
Period	24	25	26	27	28	29	30	31	32	33	34	35
Temperature	20.9	17.4	17.1	17.5	16.1	15.0	14.0	12.3	10.9	9.8	9.5	9.0
Period	36	37	38	39	40	41	42	43	44	45	46	47
Temperature	10.3	9.0	11.0	11.6	12.6	11.5	11.9	16.5	15.3	18.7	19.8	21.7

From the figures, we can see that second approach almost always provides better solutions. This is because in the second approach, the decision about what to do in every period is always based on the new information.

## 4 Conclusion

It is clear from the illustrative examples that improved profits can be achieved using the more accurate seasonal data now available from the latest climate models. To utilise these data we developed a new linear programming model to determine optimal crop planning strategies. This model keeps track of the accumulated heat units for each crop. It uses the rolling horizon approach where the planning problem is re-solved at the beginning of each period using the current state of the system and the latest forecast data. The state of the system at any time comprises what crops are currently growing and their accumulated heat units (and hence their time to maturity).

In this study, temperature is the only meteorological variable used to predict harvest time. However, the model can be extended to cover other factors such as rainfall and solar exposure using an approach similar to the concept of “accumulated heat units”.
